# Lovastatin Differentially Regulates α7 and α4 Neuronal Nicotinic Acetylcholine Receptor Levels in Rat Hippocampal Neurons

**DOI:** 10.3390/molecules25204838

**Published:** 2020-10-20

**Authors:** Virginia Borroni, Constanza Kamerbeek, María F. Pediconi, Francisco J. Barrantes

**Affiliations:** 1 Instituto de Investigaciones Bioquímicas de Bahía Blanca, Bahía Blanca 8000, Argentina; mvirborroni@gmail.com (V.B.); ckamerbeek@criba.edu.ar (C.K.); 2 Laboratory of Molecular Neurobiology, Institute for Biomedical Research, UCA–CONICET, Faculty of Medical Sciences, Catholic University of Argentina, Av. Alicia Moreau de Justo, Buenos Aires 1600 C1107AAZ, Argentina

**Keywords:** nicotinic acetylcholine receptor, cholesterol, hippocampal neurons, upregulation, homeostatic regulation, Alzheimer disease

## Abstract

Neuronal α7 and α4β2 are the predominant nicotinic acetylcholine receptor (nAChR) subtypes found in the brain, particularly in the hippocampus. The effects of lovastatin, an inhibitor of cholesterol biosynthesis, on these two nAChRs endogenously expressed in rat hippocampal neuronal cells were evaluated in the 0.01–1 µM range. Chronic (14 days) lovastatin treatment augmented cell-surface levels of α7 and α4 nAChRs, as measured by fluorescence microscopy and radioactive ligand binding assays. This was accompanied in both cases by an increase in total protein receptor levels as determined by Western blots. At low lovastatin concentrations (10–100 nM), the increase in α4 nAChR in neurites was higher than in neuronal cell somata; the opposite occurred at higher (0.5–1 µM) lovastatin concentrations. In contrast, neurite α7 nAChRs raised more than somatic α7 nAChRs at all lovastatin concentrations tested. These results indicate that cholesterol levels homeostatically regulate α7 and α4 nAChR levels in a differential manner through mechanisms that depend on statin concentration and receptor localization. The neuroprotective pleomorphic effects of statins may act by reestablishing the homeostatic equilibrium.

## 1. Introduction

Nicotinic acetylcholine receptors (nAChR) are prototypic members of the pentameric ligand-gated ion channel (pLGIC) superfamily [1]. Upon binding to the nAChR, acetylcholine—the natural ligand—promotes the opening of this ion channel, formed by five polypeptide subunits organized pseudo-symmetrically around a central pore [2]. In the central nervous system, nAChRs are present as homomeric or heteromeric receptors. The most abundant homomeric nAChR species in the central nervous system is the α7 nAChR, whereas the majority of the heteromeric nAChRs result from the combination of α4 and β2 subunits [3].

The homomeric α7 subtype plays a crucial role in various cognitive functions, including learning and memory [4,5]. There is also substantial experimental evidence supporting the notion that α7 nAChRs are critically involved in the pathogenesis of Alzheimer disease (AD) [6,7,8]. They colocalize with amyloid plaques [9], and the amyloid β peptide (Aβ) binds to α7 nAChRs [9], probably leading to internalization of Aβ by endocytosis with the ensuing build-up of intracellular Aβ [10,11]. α7 nAChR-expressing neurons are the most susceptible to AD neuropathology; both α7 nAChR binding sites and α7 nAChR protein levels are reduced in the brains of AD patients [6], influencing the neuroinflammation associated with AD [6]. Currently, it is not clear whether downregulation or upregulation of α7 nAChRs is related to the pathogenesis of AD. It is likely that interaction of this receptor with Aβ peptides contributes to the pathogenic mechanisms of cholinergic dysfunction [12].

The stability of the nAChR at the cell surface is key to the correct functioning of the cholinergic synapse. Cholesterol in particular is necessary for the maintenance of nAChRs at the plasmalemma and for ion translocation [4]. Previous studies have shown that cholesterol levels modulate the trafficking, membrane domain localization, and function of muscle-type nAChR heterologously expressed in CHO-K1/A5 cells and in endogenously expressed nAChR in C2C12 myotubes [13,14,15,16,17,18,19]. Little is known, however, on the effects of cholesterol modulation on neuronal nAChR. In hippocampal slices treated with simvastatin, α7 nAChR activity was potentiated without changes in the agonist sensitivity or desensitization kinetics. Enhancement of α7 nAChR delivery to the neuronal surface was proposed to be the mechanism behind this phenomenon [20]. In *Xenopus laevis* oocytes expressing the neuronal α7, α4β2, muscle-type, and electric fish electroplaque nAChRs, different degrees of inhibition were obtained by changing the cholesterol/phospholipid ratio in the membrane [21]. Therefore, as with muscle nAChR, it appears that neuronal nAChRs may also be modulated by cholesterol, although further research is required to understand the mechanism of this modulation.

Interestingly, disruption of cholesterol homeostasis has been associated with AD pathogenesis [22,23,24]. Early epidemiological studies reported a lower risk of dementia in patients under statin treatment [25,26] and, more recently, a combination of statins and antihypertensive drugs was shown to be more effective in reducing the risk of AD and related dementias [22]. Statins reduce cholesterol by inhibiting its biosynthesis at a critical rate-limiting step in the mevalonate pathway, i.e., by blocking the activity of HMG-CoA (5-hydroxy-3-methylglutaryl coenzyme A) reductase in the liver. Moreover, significant levels of statins were detected in mouse brain after chronic oral administration, strongly indicating that statins cross the blood–brain barrier [27]. Statins have pleiotropic effects on brain cells, some of which are not related to inhibition of cholesterol synthesis. These include changes in gene expression, neurotransmitter receptor function, neuronal membrane morphology, neurotransmitter release, and cell viability (see a recent review in [28]). The aim of this study was to characterize the effect of chronic lovastatin treatment on cellular aspects of α7- and α4-containing nAChRs. We found that lovastatin treatment augments surface expression levels, as well as total expression of α7 and α4 nAChRs, and that these increases depend on the lovastatin dose and receptor membrane localization.

## 2. Results

### 2.1. Chronic Lovastatin Treatment Reduces Cholesterol Levels in Cultured Hippocampal Neurons

**Figure 1:**
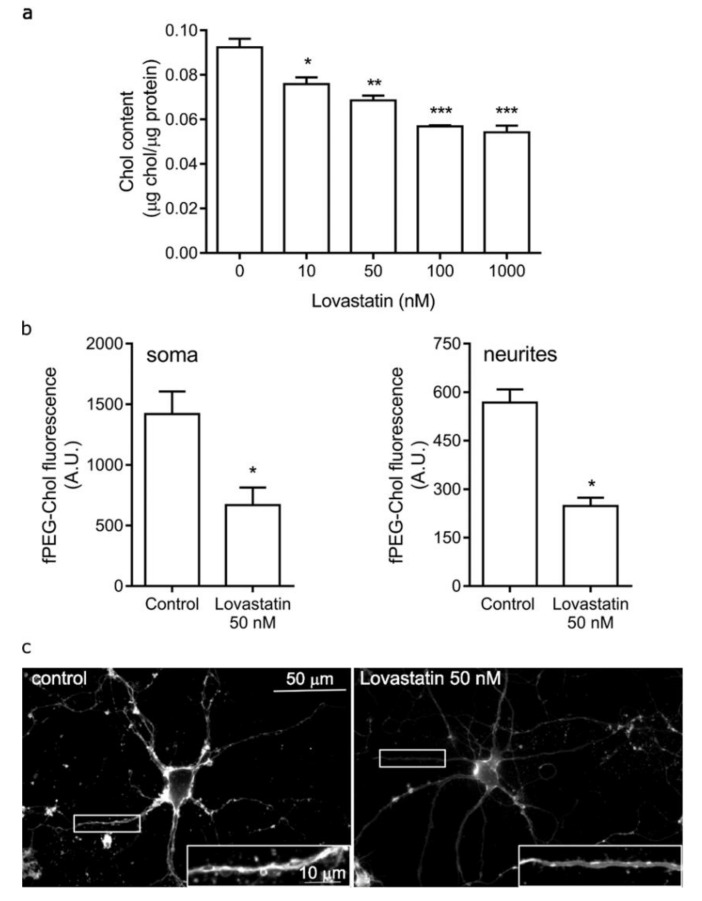
Lovastatin treatment reduced total and surface cholesterol levels in cultured hippocampal neurons. (**a**) Cultured hippocampal neurons were treated with different lovastatin concentrations for 14 days and, at the end of the incubation, total cholesterol levels were measured. (**b**) Cultured hippocampal neurons were treated with 50 nM lovastatin for 14 days or left untreated (control). At the end of the incubation, surface cholesterol was identified with the fluorescent analogue fluorescein ester of polyethylene glycol-derivatized cholesterol (fPEG-Chol). Neuronal cells were imaged and fluorescence from the soma and neurites was quantified. (**c**) Neurons treated as in (**b**) showing the different regions analyzed. Scale bar: 50 µm, inset: 10 µm. Data represent the mean ± SD of at least three independent experiments. * *p* ˂ 0.01, ** *p* ˂ 0.0025, *** *p* ˂ 0.001.

Lovastatin reduces cholesterol levels by inhibiting 3-hydroxy-3-methylglutaryl coenzyme A (HMG-CoA) reductase, the key rate-limiting enzyme in cholesterol biosynthesis. Orally administered lovastatin is able to cross the blood–brain barrier and reach the brain [28]. In primary neuronal cell cultures, the drug has direct accessibility to the target, and the dose–response curves are an accurate representation of the statin concentration in the medium, with sufficient availability and no dilution or barrier effects. In order to assess the effect of chronic lovastatin treatment on the distribution and levels of α7- and α4-containing nAChRs in neuronal cells, we incubated neurons in primary cultures with different lovastatin concentrations for up to 14 days. Importantly, the expression of nAChRs in hippocampal neurons reaches a stable plateau at day 14–15 in culture [29]. We found that lovastatin treatment significantly reduced total cholesterol levels in cultured neurons in a dose-dependent manner, at all concentrations tested (Figure 1a). We also determined the changes in cell-surface cholesterol levels by measuring the fluorescence intensity of the fluorescein ester of polyethylene glycol-derivatized cholesterol (fPEG-Chol), a cholesterol fluorescent analogue that does not cross the plasma membrane. As shown in Figure 1b,c, surface cholesterol levels were reduced in neurons treated with 50 nM lovastatin. This reduction was larger than that observed in total cholesterol levels (Figure 1a). However, the entire neuronal surface was affected similarly by lovastatin treatment. We did not observe differences in surface cholesterol levels between soma and neurites.

**Figure 2:**
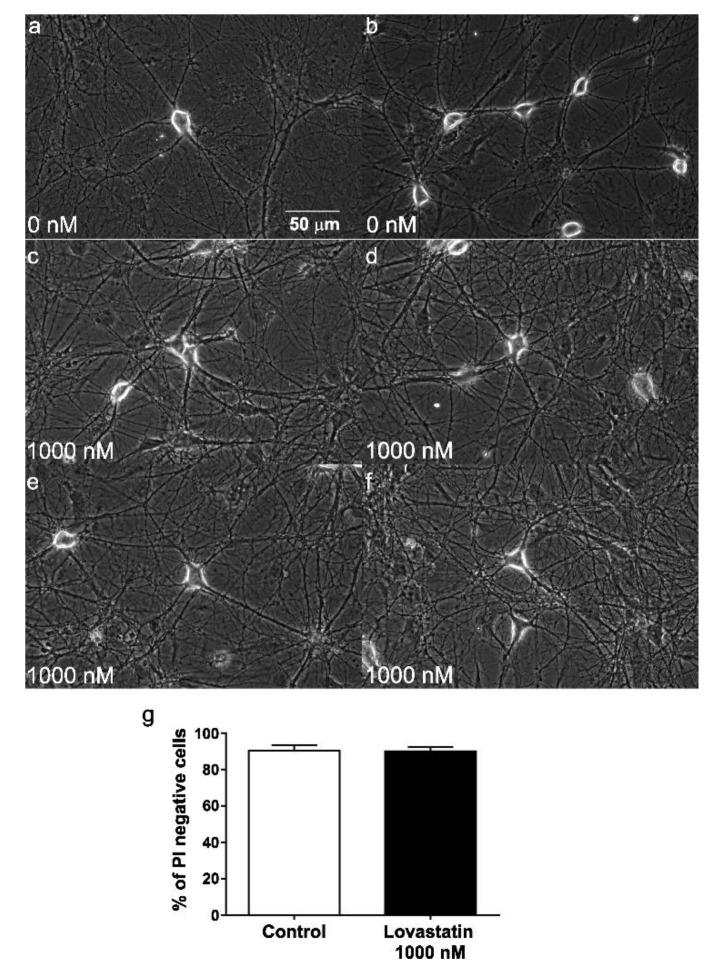
Two independent assays to assess neuronal cell viability of lovastatin treatment. (**a**) In the example shown, cultured hippocampal neurons were treated (**c**–**f**) with the maximal concentration (1000 nM) of lovastatin or with vehicle in the culture medium (**a**,**b**) for a total of 14 days. Wide-field images of typical cultures. (**g**) In the parallel fluorescence assay, neurons were stained with the probe propidium iodide (PI) and imaged. The percentage of PI-negative cells was counted and used for comparison with vehicle-treated control cells.

Importantly, lovastatin applied at a concentration of 1000 nM for 14 days did not reduce neuronal viability (Figure 2). As shown in Figure 2, the wide-field images of treated cultures (c–f) were indistinguishable from untreated control cells (a,b). There were no signs of neuronal damage i.e., loss of integrity of the membrane, shrinkage or vacuolation of the soma, and/or disruption of neurites. Moreover, the percentage of propidium iodide-negative cells in treated and control cultures (control 90% ± 3% vs. treated with 1000 nM lovastatin (90 ± 2 %, *p* = 0.9955, *n* = 3) did not differ at this concentration (Figure 2g). Since the highest concentration tested did not produce any deleterious effects on the neuronal cell cultures, it is reasonable to assume that lower concentrations did not compromise neuronal viability either.

### 2.2. Chronic Lovastatin Treatment Increases α7 nAChR Levels in Cultured Hippocampal Neurons

**Figure 3:**
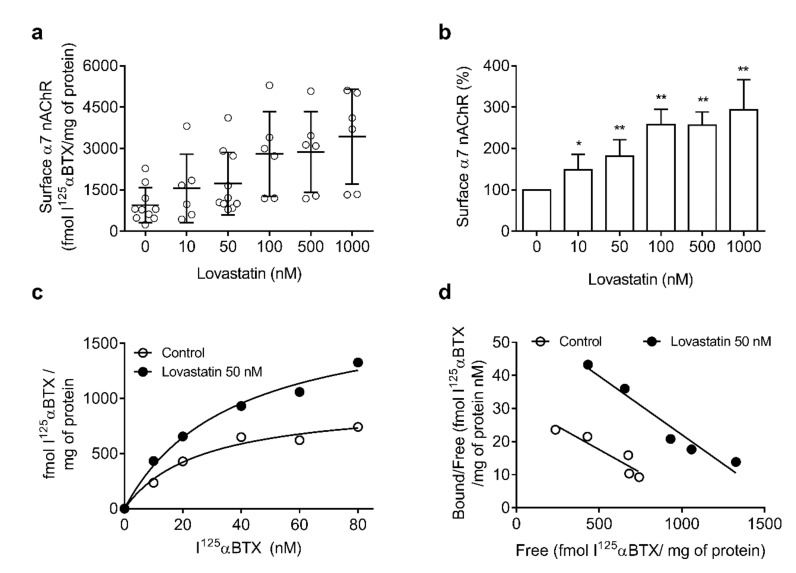
Lovastatin treatment increases surface α7 nicotinic acetylcholine receptor (nAChR) levels in cultured hippocampal neurons. (**a**) Cultured hippocampal neurons were treated with different lovastatin concentrations for a total of 14 days. At the end of the incubation, neurons were washed, fixed with paraformaldehyde (PF) for 15 min, and labeled with I^125^-α-bungarotoxin (αBTX). Neurons were washed and specific I^125^-αBTX binding was measured. Data were obtained from at least six experiments (*n* = 6–17) and represented as a scatter plot to show the variability. (**b**) Normalized data obtained in (**a**), * *p* ˂ 0.01 ** *p* ˂ 0.0025. (**c**,**d**) Representative saturation curve and Scatchard plot of data obtained as with 50 nM lovastatin.

We next evaluated α7 nAChR surface levels upon lovastatin treatment by performing binding experiments with the radioactive ligand I^125^-α-bungarotoxin (αBTX) and fluorescence microscopy experiments with Alexa^488^-αBTX. This toxin is a quasi-irreversible antagonist that binds with high affinity to α7 nAChR but not α4 nAChR, thus providing a specific probe for measuring the α7-type receptor. As shown in Figure 3, the surface area of α7 nAChRs in 14 days in vitro neurons was highly variable among experiments (200–2200 fmol/mg protein, *n* = 17). The variability persisted in lovastatin-treated samples, with a statistically significant increase in α7 nAChR surface levels. This could be related to the fact that, although pyramidal neurons are the main cell type in the hippocampus, several other types of neuronal cells, particularly interneurons, are also present [30]. The proportion of different neuronal populations may have also varied between different cultures, resulting in the relatively high variability of the data. In fact, the increase became clearly apparent upon normalization of the data (Figure 3b) and was not accompanied by changes in the apparent affinity of α7 nAChR for αBTX (Figure 3c,d), as shown in the Scatchard plots.

### 2.3. Chronic Lovastatin Treatment Increases Surface α7 and α4 nAChR Levels Differentially in Soma and Neurites of Cultured Hippocampal Neurons

Since neurons are highly polarized cells, it was of interest to study whether the changes in α7 nAChR and α4 nAChR levels upon chronic statin treatment differed between soma and neurites (dendrites and axon). To this end, we performed fluorescence microscopy experiments.

**Figure 4:**
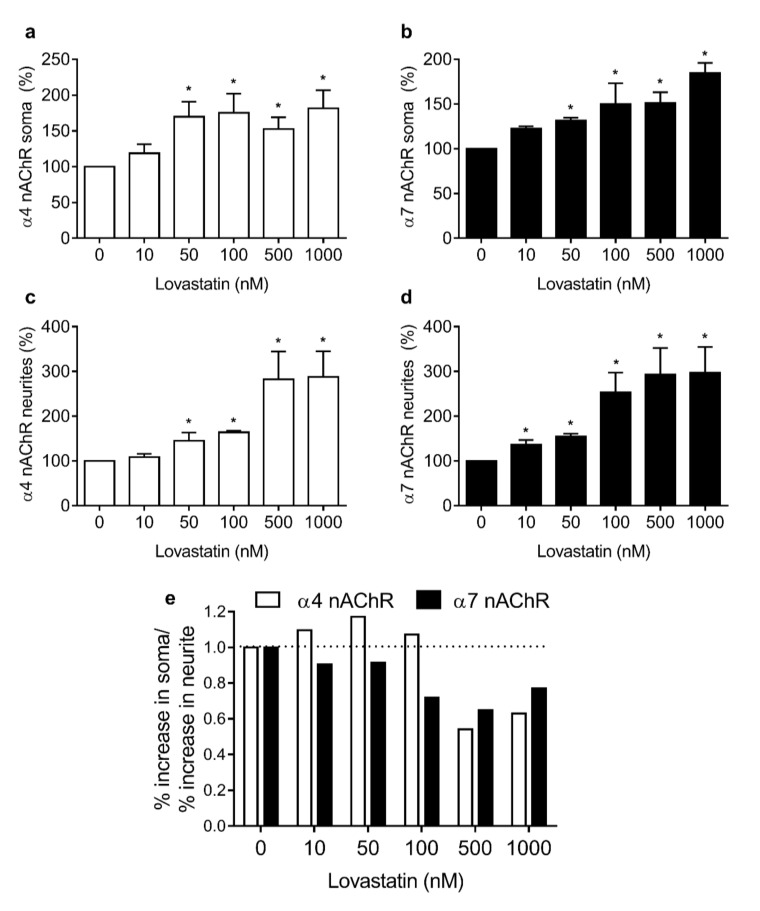
Lovastatin treatment increases surface α4 and α7 nAChR levels differentially in soma and neurites. Cultured hippocampal neurons were treated with different lovastatin concentrations for 14 days. At the end of the incubation neurons were washed, fixed with PF for 15 min, and labeled with Alexa Fluor^488^-BTX and mAb-299 (a primary monoclonal antibody against the α4 subunit) and Alexa Fluor^555^- secondary antibody. Neurons were washed with phosphate-buffered saline (PBS) and imaged by epifluorescence microscopy. Fluorescence corresponding to Alexa Fluor^555^ (**a**,**c**) and Alexa Fluor^488^-BTX (**b**,**d**) in soma (upper panel) and neurites (lower panel) was quantified and normalized. Data represent the mean ± SD from at least three independent experiments. * *p* ˂ 0.01. (**e**) Relative increase in Alexa Fluor^555^ (empty bars) and Alexa Fluor^488^-BTX (black bars) fluorescence in soma and neurites.

Neurons grown for 14 days in vitro in the absence or presence of increasing concentrations of lovastatin were labeled with Alexa^488^-αBTX and mAb-299, a primary monoclonal antibody against the α4 subunit. After labeling with AlexaFluor^555^-labeled secondary antibody, neurons were washed and imaged. Fluorescence associated with AlexaFluor^488^-αBTX and AlexaFluor^555^-secondary antibody was quantified in the plasma membrane of the neuronal somas and in neurites, as shown in Figure 4. Lovastatin treatment increased surface α4 and α7 nAChR levels differentially in soma and neurites. The lovastatin-induced increase in α4 nAChR levels was already apparent at lovastatin concentrations as low as 50 nM in both somata and neurites (Figure 4a,b). At higher concentrations of the drug, the increase in neurites was greater than in the soma, reflecting a relative enrichment of α4 nAChR in neurite membranes (Figure 4a,b). Lovastatin treatment also increased α7 nAChR levels in both soma and neurites, but the increase in neurites was already apparent at lower concentrations (10 nM) than in soma (Figure 4b,c).

The lovastatin-induced increase in α4 nAChR levels was already apparent at lovastatin concentrations as low as 50 nM in both somata and neurites (Figure 4a,b). At higher concentrations of the drug, the increase in neurites was greater than in the soma, reflecting a relative enrichment of α4 nAChR in neurite membranes (Figure 4a,b).

Lovastatin treatment also increased α7 nAChR levels in both soma and neurites, but the increase in neurites was already apparent at lower concentrations (10 nM) than in soma (Figure 4b,c). When the ratio of the increment percentage in somata and neurites was calculated for each nAChR subtype, differences became more obvious. At low lovastatin concentrations (10–100 nM), the increase in α4 nAChR in soma was clearly higher than in neurites (ratio > 1); however, at high lovastatin concentrations, the opposite was apparent (ratio < 1) (Figure 4d). This suggests that the α4 nAChR present in soma is more sensitive to cholesterol modification than the α4 nAChR present in neurites. In contrast, the α7 nAChR present in neurites is more sensitive to cholesterol depletion than somatic α7 nAChR (Figure 4d).

**Figure 5:**
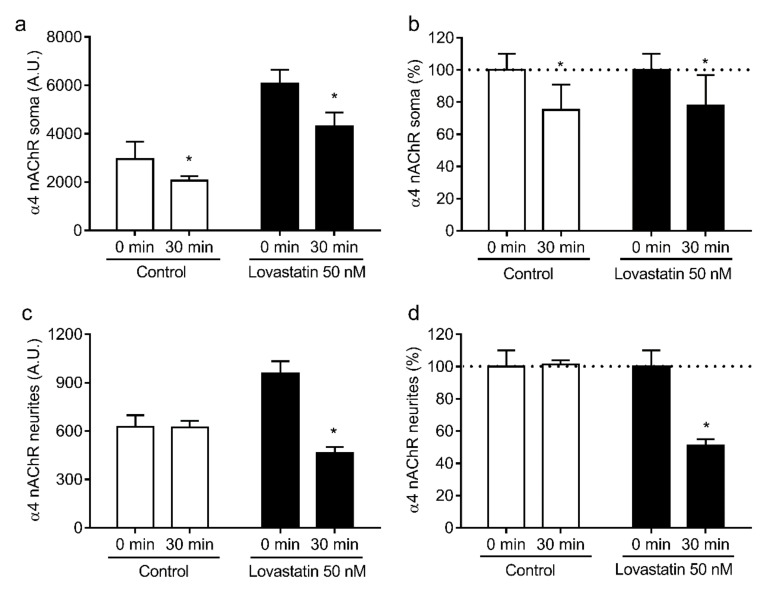
Lovastatin treatment affects surface α4 nAChR internalization differentially in soma and neurites. Cultured hippocampal neurons were treated with 50 nM lovastatin for 14 days or left untreated (control). At the end of the incubation, neurons were washed, labeled with mAb-299 for 1 h at 4 °C, and then transferred to an incubation bath at 37 °C for 30 min. After the incubation, neurons were fixed and α4 nAChRs remaining at the surface were revealed by labeling with Alexa Fluor^555^-secondary antibody. Fluorescence was quantified by fluorescence microscopy in soma (**a**,**b**) and neurites (**c**,**d**) and expressed in arbitrary units (AU) or normalized against the fluorescence obtained without incubation at 37 °C (0 min). Data represent the mean ± SD from at least three independent experiments. * *p* ˂ 0.01.

**Figure 6:**
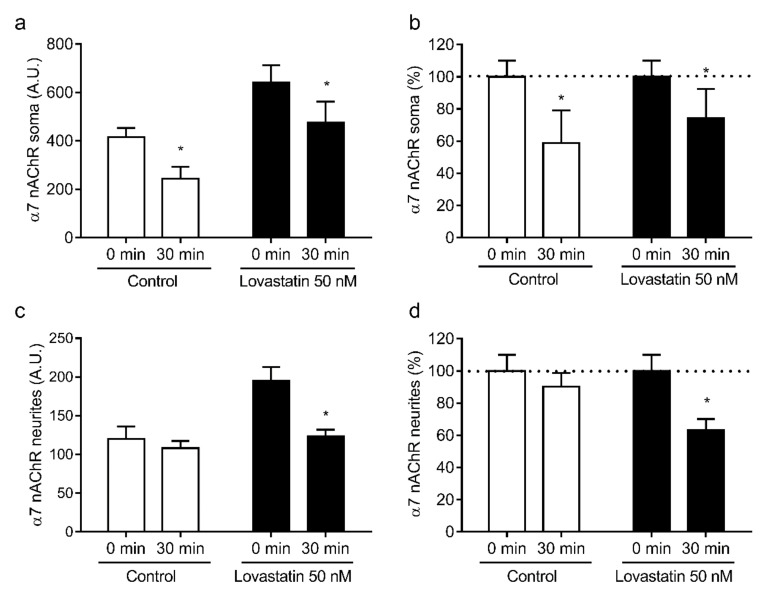
Lovastatin treatment affects surface α7 nAChR internalization differentially in soma and neurites. Cultured hippocampal neurons were treated with 50 nM lovastatin for a total of 14 days or left untreated (control). At the end of the incubation, neurons were washed, labeled with biotin-BTX for 1 h at 4 °C, and then transferred to an incubation bath at 37 °C for 30 min. After the incubation, neurons were fixed, and α7 nAChRs remaining at the surface were revealed by labeling with Alexa^488^-streptavidin. Fluorescence was quantified by fluorescence microscopy in soma (**a**,**b**) and neurites (**c**,**d**) and expressed in arbitrary units (AU) or normalized against the fluorescence obtained without incubation at 37 °C (0 min). Data represent the mean ± SD from at least three independent experiments. * *p* ˂ 0.01.

These differences between somata and neurites could result from differences in the stability of the nAChRs present in each neuronal compartment. To address this possibility, we studied the internalization rate of nAChR in neuronal somata and neurites upon treatment with 50 nM lovastatin. We chose this concentration because it produces a clear decrease in cholesterol levels well below saturation, which occurs only at higher doses. Neurons were labeled with the anti-α4 monoclonal antibody mAb-299, incubated at 37 °C for 30 min, and labeled with a secondary antibody at the end of the incubation period. Using this labeling protocol, surface fluorescence was proportional to nAChR levels remaining at the neuronal surface, i.e., not internalized by endocytic mechanisms. As observed previously, lovastatin treatment increased surface α4 nAChR levels, and this increment was higher in the neuronal soma. Interestingly, in control neurons, α4 nAChRs were internalized in the soma at a faster rate than in the neurites (75.2% ± 15.9% α4 nAChR/30 min vs. 101.2% ± 2.7% α4 nAChR/30 min, *p* ≤ 0.001, Figure 5b,d, 30 min control). Moreover, the internalization rate of α4 nAChR in soma was not affected by lovastatin treatment (75.2% ± 15.9% α4 nAChR/30 min vs. 77.9% ± 19.0% α4 nAChR/30 min *p* = 0.791, Figure 5b, 30 min control vs. lovastatin), whereas, in neurites, α4 nAChR internalization was accelerated upon cholesterol depletion (101.2% ± 2.7% α4 nAChR/30 min vs. 51.1% ± 3.9% α4 nAChR/30 min, *p* ≤ 0.001, Figure 5d, 30 min control vs. lovastatin). However, the internalization rate of α7 nAChR present in the soma was not affected by cholesterol depletion (58.8% ± 20.2% α7 nAChR/30 min vs. 74.2% ± 18.2% α7 nAChR/30 min, *p* = 0.228, Figure 6b, 30 min control vs. lovastatin), whereas α7 nAChR internalization was accelerated in neurites (63.2% ± 6.9% α7 nAChR/30 min vs. 90.3% ± 8.4% α7 nAChR/30 min, *p* ≤ 0.001, Figure 6d, 30 min control vs. lovastatin).

### 2.4. Chronic Lovastatin Treatment Increases α4 and α7 nAChR Total Levels in Cultured Hippocampal Neurons

**Figure 7:**
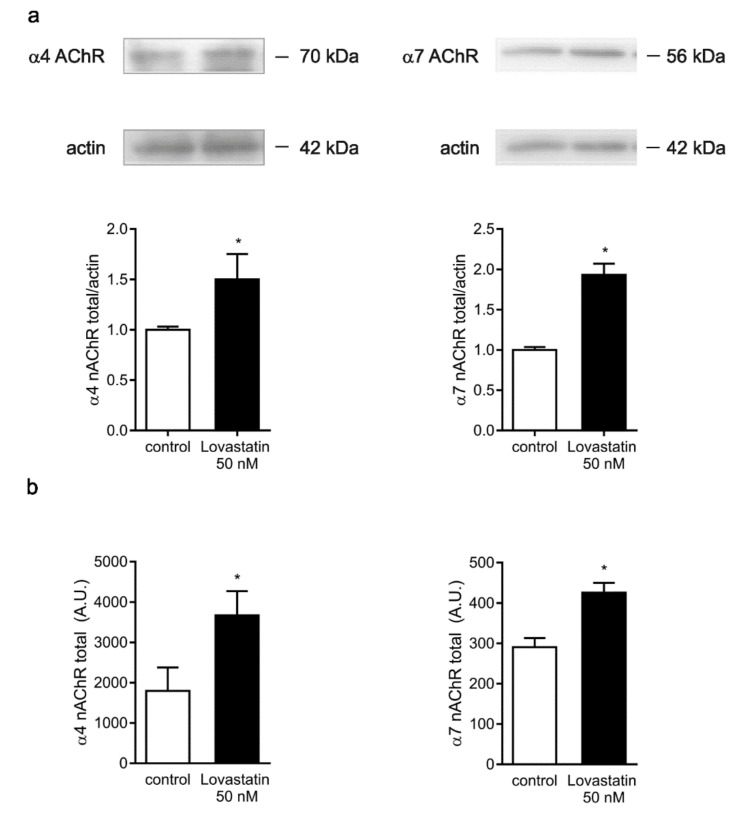
Lovastatin treatment increased total α7 and α4 nAChR levels. (**a**) α7 and α4 nAChR subunits revealed in immunoblots of cultured hippocampal neurons treated with 50 nM lovastatin for 14 days or left untreated (control). Actin was used as loading control. Lower panel: quantification of α7 and α4 nAChR subunits against actin. Data represent the mean ± SD from at least three independent experiments. (**b**) Cultured hippocampal neurons were treated with 50 nM lovastatin for 14 days or left untreated (control). At the end of the incubation, neurons were fixed, permeabilized, and labeled with Alexa Fluor^488^-BTX and mAb-299 for 1 h at room temperature. After washing, neurons were incubated with Alexa Fluor555-secondary antibody for 2 h, washed with PBS, and imaged; then, fluorescence was quantified. Data represent the mean ± SD from at least three independent experiments. * *p* ˂ 0.01.

The increase in cell-surface α4 and α7 nAChRs could also result from an increase in receptor synthesis and membrane insertion. To test this hypothesis, we measured total (internal + surface) α7 and α4 nAChR levels in permeabilized neurons treated with 50 nM lovastatin using two independent assays: fluorescence microscopy and Western blotting. The two methods employed concurrently demonstrated that lovastatin treatment increased total α7 and α4 nAChR levels with respect to control, untreated neurons (Figure 7).

## 3. Discussion

Although the brain accounts for only 2% of the total weight of our organism, its cholesterol content amounts to ~25% of the total [31]. Another peculiarity of brain cholesterol is that its content is regulated autonomously due to the impermeability of the blood–brain barrier to macromolecules involved in cholesterol transport, such as the plasma lipoproteins [30]. Here, we found that chronic lovastatin treatment reduced total cholesterol levels in neuronal cells, even at low doses. More relevant, the neuronal membrane was evenly depleted in cholesterol. Interestingly, the results obtained with neuronal cells in culture correlate well with those from in vivo studies. Chronic administration of hydrophobic statins such as lovastatin or simvastatin and the more hydrophilic statin, pravastatin, specifically reduces neuronal membrane cholesterol levels in vivo, as measured in synaptosomal membranes and brain membranes of mice. In particular, lovastatin and pravastatin were shown to significantly reduce the cholesterol content of cholesterol-rich microdomains in the exofacial leaflet of the membrane [32,33]. However, it is worth mentioning that these drugs have effects on cells beyond inhibition of cholesterol biosynthesis [28].

Cholesterol exerts effects on multiple aspects of synaptic transmission [34], both presynaptically, acting on neurotransmitter vesicle fusion [35], and postsynaptically, modifying neurotransmitter receptor diffusion and domain localization in the postsynaptic membrane [36], endosomal dynamics [37], and receptor ion translocation [38]. Therefore, it is unlikely that the nAChR constitutes the only target of lovastatin treatment.

Life-long synaptic activity and accumulated metabolic stress appear to contribute to a moderate but irreversible loss of membrane cholesterol in the aging brain [39,40]. It has been speculated that this loss underlies neuronal dysfunctions and the cognitive deficits present at this stage of life [41,42,43]. It has also been surmised that dysregulation of cholesterol metabolism is a major factor in neurological diseases accompanied by cognitive dysfunction, such as Alzheimer disease [44,45] (reviewed in [23]), Parkinson disease [46], and Huntington disease [47]. Epidemiological studies have suggested that individuals treated with statins have a lower risk of developing Alzheimer disease [25,28,48]. In this study, we demonstrated that chronic statin treatment increased both α7 and α4 nAChR. At low lovastatin doses, there was an enrichment of α4 nAChR in neuronal somata, whereas, at high doses, the increase in α4 in neurites was more prominent. Interestingly, α7 nAChRs in neurites (axon + dendrites) were more susceptible to lovastatin treatment. At all doses tested, the increase in neurite α7 was higher than in somatic α7. Neurites are the site where most of the synaptic transmission occurs. Thus, changes in the relative amount of neurotransmitter receptors, as shown in this work, may have profound effects on synaptic responses. Indeed, hippocampal slices treated with simvastatin showed a significant potentiation of α7 nAChR activity without changes in agonist sensitivity or in the kinetics of desensitization [20]. Our results suggest that the mechanism behind this phenomenon is an increase in α7 nAChR delivery to the neuronal surface. Given the marked cholinergic deficit in AD [12,49], the neuroprotective effect mediated by chronic statin treatment could be related to the increase in surface nAChR and the consequent maintenance/restoration of cholinergic activity.

The mechanism via which lovastatin treatment increases nAChR levels may involve multiple stages. The internalization rate of neuronal nAChRs varies according to receptor type and localization. Whereas the internalization rate of α4 in neuronal cell somata was not affected at 50 nM lovastatin, it increased in neurites, and this phenomenon may explain the relative increase of α4 nAChR in somata at this lovastatin concentration. The α7 nAChR internalization rate in neuronal somata was slightly hindered at 50 nM lovastatin, whereas, in neurites, it was markedly augmented. Interestingly, the global result was a net increase in neurite α7 nAChR. Preferential neurite insertion of newly synthesized receptors may explain the differences. Moreover, total nAChR levels were increased, and this may also contribute to the gain in surface nAChR receptor pool. This is in agreement with the results of Roentsch and coworkers [50], who demonstrated that a series of statins, including lovastatin, augmented the expression of α7 subunit messenger RNA (mRNA) in SH-SY5Y cells. Similar results were obtained in hippocampal slices by Chen et al. [51], although, in this case, no increase in α4 mRNA expression was observed. It is important to note that protein expression was not measured in the latter study. An increase in α4 nAChR protein stability may explain the increase observed here. nAChRs are very sensitive to membrane cholesterol levels; ion translocation properties, membrane domain localization, rate and mechanism of internalization, and exocytic trafficking are strongly dependent on membrane cholesterol in muscle nAChR [13,14,15,16,19]. The results presented in this paper indicate that the cholesterol dependence may be a hallmark not only of the muscle-type nAChR but of the entire nAChR family.

In addition to lowering cholesterol levels, statins also have pleiotropic effects, including immunomodulatory [52], antioxidant, and anti-inflammatory [28,53,54] effects that may be related to their purported beneficial effects on Alzheimer disease. The homeostatic modulation of α7 and α4 nAChRs levels by cholesterol could be a hitherto ignored mechanism via which statins exert their neuroprotective action.

## 4. Materials and Methods

### 4.1. Materials

2-Methyl-1,2,3,7,8,8a-hexahydro-3,7-dimethyl-8-[2-(tetrahydro-4-hydroxy-6-oxo-2*H*-pyran-2-yl)ethyl]-1-naphthalenyl ester butanoic acid), “lovastatin”, α-bungarotoxin (αBTX), bovine serum albumin (fraction V, cell culture tested), ovalbumin, chloramine T, sodium metabisulfite, rat anti-nAChR α4 subunit monoclonal antibody mAb-299, glutamine, propidium iodide, sodium pyruvate, and poly-l-lysine hydrobromide were purchased from Sigma Chemical Company (St. Louis, MO, USA). [^125^I]iodine was obtained from Perkin Elmer (Wellesley, MA, USA). Alexa Fluor^488^-conjugated αBTX (Alexa Fluor^488^-αBTX), biotin-αBTX, and Alexa Fluor^633^-streptavidin were purchased from Molecular Probes (Eugene, OR, USA). Neurobasal, trypsin, cytosine arabinoside, N2, and B27 supplements were from Invitrogen (Carlsbad, CA, USA). Rabbit ab-23832 anti-α7 nAChR polyclonal antibody and anti-actin were purchased from AbCam (Eugene, OR, USA). Mouse monoclonal antibody anti-tubulin was purchased from BD Biosciences (San Jose, CA, USA). Horseradish peroxidase (HRP)-conjugated goat anti-rabbit and goat anti-mouse secondary antibodies were purchased from Santa Cruz Biotechnology, Inc. (Santa Cruz, CA, USA). The fluorescein ester of polyethylene glycol-derivatized cholesterol (fPEG-Chol) was a gift from Prof. T. Kobayashi and Dr. Satoshi B. Sato, and anti-α7 nAChR monoclonal antibodies mAb-306 and mAb-307 were gifts from Dr. Jon Lindstrom, University of Pennsylvania.

### 4.2. Hippocampal Cultures

Dissociated neuronal cultures were prepared from hippocampi of embryonic day 19, as previously described [55]. Briefly, brain tissue was treated with 0.25% trypsin in Hanks’ solution at 37 °C for 15 min. A single-cell solution was prepared in Neurobasal (NB) medium containing 2 mM glutamine, 10 µM sodium pyruvate, 100 units/mL penicillin, and 100 µg/mL streptomycin (NB1X) with 10% (*v*/*v*) horse serum. Cells were seeded on coverslips coated with 0.1 mg/mL poly-l-lysine hydrobromide at a density of 30,000 cells/cm^2^. After 2 h, the medium was changed to NB/N2 (NB1X with 1 g/L ovalbumin; N2 and B27 serum-free supplements). On day 3, cytosine arabinoside was added to inhibit glial development. On the basis of morphological characteristics, it was estimated that >90% of the cells in the primary culture were neurons. Lovastatin treatment started at 6 days in vitro.

### 4.3. Protein Content

Protein content was determined using the method of Lowry et al. (1951) upon solubilization of cells with 0.1 N NaOH, using bovine serum albumin (BSA) as a standard.

### 4.4. Cholesterol Determination

Total cholesterol content was measured using a commercial kit (Colestat, AA, Wiener, Rosario, Argentina) following manufacturer’s instructions.

Surface cholesterol was evaluated by incubation of the neurons with 1 μM fPEG-Chol in phosphate-buffered saline (PBS) at 4 °C, followed by washing with PBS and observation under the microscope.

### 4.5. αBTX Radioiodination

For all binding experiments, the specific ligand [^125^I]-α-bungarotoxin ([^125^I]-αBTX) was prepared in our laboratory. To this end, 80 μg of αBTX was mixed with 2 µL of chloramine T (40 mg/mL) and with 0.66 mCi [^125^I]Na for 2 min at room temperature. The reaction was stopped by addition of 2 µL of sodium metabisulfite solution at a final concentration of 40 mg/mL. The mixture was subsequently applied to a G25 medium Sephadex (Pharmacia) column where the resulting iodinated [^125^I]-αBTX was separated from the free iodine. The specific activity of the [^125^I]-αBTX obtained amounted to ~50 µCi/mmol.

### 4.6. Equilibrium [^125^I]-αBTX Binding Studies

Surface nAChR expression was determined by incubating 70–80% confluent neuronal cells with increasing concentrations (10–60 nM) of [^125^I]-αBTX in the cell culture medium at 25 °C for 50 min. After incubation, dishes were washed twice with phosphate buffer, and cells were removed and collected by addition of 1.5 mL of 0.1 N NaOH. Radioactivity was measured in a gamma counter with 80% efficiency. Nonspecific binding was determined from the radioactivity remaining in the dishes after preincubation of cells with 50 mM carbamoylcholine chloride for 1 h before addition of [^125^I]-αBTX. Non-specific binding amounted to <10% in all experiments. Determination of the total pool of nAChR was carried out upon permeabilization of cells with 0.5% saponin. The nAChR intracellular pool was calculated as the difference between the total and [^125^I]-αBTX surface binding sites.

### 4.7. Cell-Surface α7 and α4 nAChR Labeling

Neurons were fixed with 2% paraformaldehyde for 15 min and subsequently incubated overnight with mAb-299 in PBS/BSA at 4 °C. At the end of the incubation neurons, were washed thrice with PBS and incubated with Alexa Fluor^488^-αBTX or Alexa Fluor^555^-secondary antibody for 2 h at room temperature.

### 4.8. Internalization Assay

Neurons were incubated with mAb-299 or biotin-αBTX in Neurobasal medium for 1 h at 4 °C, and then transferred to a thermostatic bath at 37 °C for an additional 30 min period. At the end of the incubation, neurons were fixed for 15 min in 2% paraformaldehyde, washed thrice with PBS, and labeled with Alexa^488^-streptavidin or Alexa Fluor^555^-secondary antibody for 2 h at room temperature.

### 4.9. Fluorescence Microscopy

Labeled neurons were examined with a Nikon Eclipse E-600 microscope. Imaging was accomplished with a SBIG model ST-7 digital charge-coupled device camera (765 × 510 pixels, 9.0 × 9.0 μm pixel size; Santa Barbara, CA, USA). The ST-7 CCD camera was driven by the CCDOPS software package (SBIG Astronomical Instruments, version 5.02). For all experiments, 40× (1.3 numerical aperture (NA)) or 100× (1.4 NA) oil-immersion objectives were used. Appropriate dichroic and emission filters were used to avoid crossover of fluorescence emission. Then, 8 bit or 16 bit TIFF images were exported for further off-line analysis.

### 4.10. Quantitative Image Analysis

Fluorescence intensities of the 8 or 16 bit images were analyzed after manually outlining regions of interest (ROI) with the software Image J (NIH, Bethesda, MD, USA). The average fluorescence intensity of a given ROI was measured, and the average fluorescence intensity of an area of the same field outside the neuron was subtracted. These measurements were undertaken on randomly chosen neurons, selected from phase-contrast images to avoid bias, for each experimental condition. For illustration purposes, images were scaled with identical parameters, and pseudo-colored according to a custom designed look-up-table (LUT).

### 4.11. Western Blotting

At the end of each treatment, neurons were harvested and lysed at 4 °C for 1 h with cell lysis buffer (20 mM 4-(2-hydroxyethyl)-1-piperazineethanesulfonic acid (HEPES); 10 mM ethylene glycol tetraacetic acid (EGTA); 5 mM β-glicerophosphate, 1% Nonidet P-40; 2.5 mM MgCl_2_) containing protease inhibitors (1 mM dithiothreitol (DTT); 2 µg/mL leupeptin; 1 µg/mL aprotinin; 1 µg/mL pepstatin; 0.1 mM phenylmethylsulfonyl fluoride (PMSF)). The neuron suspension was centrifuged at 13,200 rpm for 20 min at 4 °C. The supernatant was subsequently collected, and protein content was determined following the procedure of Lowry et al. (1951). Samples were then denatured with Laemmli buffer at 100 °C for 5 min and proteins were resolved by sodium dodecyl sulfate polyacrylamide gel electrophoresis (SDS-PAGE) on 10% polyacrylamide gels and subsequently transferred to polyvinylidene fluoride (PVDF) membranes (Millipore, Bedford, MA, USA). These membranes were blocked with 10% BSA in Tris-buffered saline/Tween-20 (TBST) buffer (20 mM Tris–HCl (pH 7.4) containing 100 mM NaCl and 0.1% (*w*/*v*) Tween-20) at room temperature for 2 h. Membranes were subsequently incubated overnight at 4 °C with a 1:1000 dilution of primary antibodies (anti-α7 nAChR, anti-α4 nAChR, and anti-actin), washed thrice with TBST, and subsequently exposed to the appropriate HRP-conjugated secondary antibody (anti-rabbit or anti-mouse) for 2 h at room temperature. Membranes were washed again three times with TBST and immunoreactive bands were detected upon exposure to the enhanced chemiluminescence reagent (ECL, Amersham Biosciences) using standard X-ray film (Kodak X-Omat AR). About 40–50 µg of proteins were loaded per lane in all experiments.

### 4.12. Viability Assays

The number of dead neurons was quantified by propidium iodide (PI) staining. Briefly, PI (2 µM) was added to control and lovastatin-treated neurons, and the culture plates were incubated for 30 min at 37 °C. The medium was then removed and the neurons were washed three times with PBS, followed by observation under an inverted fluorescence microscope with appropriate filters (excitation maximum, 535 nm; emission maximum, 617 nm) to estimate the number of PI-positive cells.

### 4.13. Data Analysis

Data were analyzed using GraphPad Prism program from GraphPad Software Inc., San Diego, CA, USA. Statistically significant differences were determined by Student’s *t*-test (two-tailed) or one-way ANOVA with Tukey post hoc test, as appropriate.
